# Glycyrrhetinic Acid Liposomes and Hyalurosomes on Spanish Broom, Flax, and Hemp Dressings to Heal Skin Wounds

**DOI:** 10.3390/molecules25112558

**Published:** 2020-05-31

**Authors:** Angela Abruzzo, Concettina Cappadone, Giovanna Farruggia, Barbara Luppi, Federica Bigucci, Teresa Cerchiara

**Affiliations:** Department of Pharmacy and Biotechnology, Alma Mater Studiorum–University of Bologna, Via San Donato 19/2, 40127 Bologna, Italy; angela.abruzzo2@unibo.it (A.A.); concettina.cappadone@unibo.it (C.C.); giovanna.farruggia@unibo.it (G.F.); federica.bigucci@unibo.it (F.B.); teresa.cerchiara2@unibo.it (T.C.)

**Keywords:** wound dressing, glycyrrhetinic acid, liposomes, hyalurosomes, cytotoxicity, Spanish Broom, flax, hemp

## Abstract

The focus of this work was to prepare Spanish Broom, flax, and hemp dressings impregnated with glycyrrhetinic acid (GA) liposomes or hyalurosomes to promote the healing process and protect the skin wounds. Vesicles were prepared by the film hydration method and characterized in terms of size, particle size distribution, ζ potential, encapsulation efficiency, in vitro release, and biocompatibility on 3T3 fibroblasts. Loaded liposomes and hyalurosomes showed nanometric size (355 ± 19 nm and 424 ± 32 nm, respectively), good size distribution (lower than 0.3), and appropriate encapsulation efficiency (58.62 ± 3.25% and 59.22 ± 8.18%, respectively). Hyalurosomes showed good stability during the storage period, which can be correlated to the negative ζ potential, and allowed a fast and complete release of GA. Preliminary biological studies revealed that both kinds of loaded vesicles were not cytotoxic and that hyalurosomes could exert a slight stimulating effect on fibroblast proliferation. Finally, in vitro release studies from the different dressings impregnated with the loaded vesicles demonstrated that a high amount of GA could be reached at the wound site after 60 min from application. In conclusion, the results suggested that the developed dressings, especially those impregnated with hyalurosomes, can be efficiently used to promote the healing process.

## 1. Introduction

In recent years, the focus of wound-care has been based on research and development of new antibacterial and anti-inflammatory wound dressings able to improve and accelerate the healing process. This process is based on four different phases including hemostasis, which occurs immediately after injury, followed by the inflammatory phase, the formations of new vessels, and finally the maturation of new tissues [1,2,3]. The first step in order to effectively heal the wound and to prevent it from any infectious agent requires the cover of the wound by an appropriate material [4]. Traditional wound dressings are textile fibers such as cotton, wool, and silk, but new wound dressing materials are an important part of the rapidly growing biomaterials industry [5].

In this regard, the aim of this study was the development of new Spanish Broom, flax, and hemp wound dressings impregnated with nanosystems containing 18-β-glycyrrhetinic acid (GA) for wound healing management. Spanish Broom, flax, and hemp are bast fibers [6] and can be considered as sustainable and biodegradable alternatives to cotton owing to their availability, renewability, and cleaner and more resilient cultivation. Cotton fibers are the most popular natural fibers, widely used in various textile industries. However, its cultivation is environmentally unsustainable owing to use of pesticides and a large amount of water [7]. Spanish Broom, flax, and hemp fibers as well as cotton are composed of cellulose fibers suitable for use in the treatment of wounds as well as in the hygiene sector, owing to their high compatibility with skin and wounds [8]. GA, a pentacyclic triterpenoid found in the *Glycyrrhiza glabra* L. liquorice roots [9], was selected as bioactive molecule with proven anti-inflammatory, antioxidant, and antimicrobial activity [10,11,12]. The inhibition of the inflammatory cascade together with the antioxidant mechanism of GA may increase the repair process rate of the damaged skin by restoring the physiological balance and tissue functionality [9]. According to Biopharmaceutical Classification System, GA is a type II drug characterized by low solubility [13,14] and, to overcome this drawback, appropriate formulative strategies such as the encapsulation of GA in nanosystems might be used. In recent years, nanoparticles [15], liposomes [16], and multifunctional nanofibers [17] have been investigated to increase drug solubility and be applied as drug carriers. Among them, liposomes have become promising drug delivery systems for skin wounds thanks to several advantages such as biocompatibility, safety, and ability to be directly applied to the wounds [18].

In order to prepare a performing dressing for wound treatment, we combined the possibility to protect skin wounds using different supporting biomaterials and to deliver at the wound site functional nanosystems able to improve the interaction with the biological substrates, thus promoting the healing process. To the best of our knowledge, this is the first study reporting the use of new dressings based on Spanish Broom, flax, and hemp impregnated with different GA-loaded vesicles. Specifically, GA-loaded liposomes or hyalurosomes were prepared and characterized for their physico-chemical and functional properties, such as size, particle size distribution, ζ potential, encapsulation efficiency, and ability to release GA. In addition, vesicle cytotoxicity and cell proliferation activity were evaluated through 3-(4,5-dimethylthiazol-2-yl)-2, 5-diphenyl tetrazolium bromide (MTT) assay and cell cycle analysis on 3T3 fibroblasts, respectively. Finally, loaded vesicles were sunk into Spanish Broom, flax, and hemp dressings and in vitro release tests were performed in order to study dressings ability to release GA over time.

## 2. Results and Discussion

In this work, GA-loaded liposomes and hyalurosomes were prepared through the film hydration method followed by extrusion and subsequently sunk into Spanish Broom, flax, or hemp gauzes, by impregnation.

GA, the major active component of licorice root extract, possesses well-documented anti-inflammatory, antimicrobial, and antioxidant properties and could represent a valid approach to treat skin wounds [9,12]. However, despite the wide spectrum of beneficial properties, the clinical application of GA has been hampered owing to its low water solubility [13,14]. In this context, the development of an adequate formulation, able to improve GA solubility, represents a key challenge for its delivery. Liposomes were selected considering their ability to straightforwardly entrap hydrophobic molecules into phospholipid bilayers and their optimal properties for skin drug delivery [19]. Furthermore, in this work, modified vesicles were also prepared by employing hyaluronic acid sodium salt, a water-soluble polymer possessing, at pH higher than its pka (≈2.9), negatively charged groups able to stabilize the vesicles [20]. In addition, sodium hyaluronate was selected on the basis of its high biocompatibility and its well-known role in skin wound healing [21].

In order to optimize the application of liposome suspensions on skin wounds, simplify self-medication, and consequently increase patient compliance, a suitable support should be adopted. In this study, taking into account the increasing request to find new biomaterials for wound treatment [22], three different types of wound dressings, Spanish Broom, flax, and hemp, were proposed as alternative to traditional cotton dressing. Spanish Broom fibers were used in our previous studies for the delivery of different active substances in the treatment of skin wounds [7,23], and our findings allowed us to propose them as an attractive and ecologically sustainable alternative for the preparation of dressings for wound care. In this work, Spanish Broom dressings were compared with flax and hemp, selected as additional materials for wound dressing on the basis of their interesting characteristics, such as the high hydrophilic nature and the capacity to absorb a large amount of water.

### 2.1. Vesicle Characterization

The main physicochemical properties of liposomes and hyalurosomes are summarized in Table 1. The presence of sodium hyaluronate allowed to obtain vesicles with a larger size with respect to liposomes (*p* < 0.05), probably owing to the polymer ability to interact with the choline groups of phosphatidylcholine and to promote the formation of bigger structures. This result was in agreement with previous findings reported by Castangia and co-workers [12], who attributed the size increase of vesicles containing hyaluronic acid to the absorption of polymer onto their surface and its intercalation between the bilayers. Moreover, the sizes of LP_GA_ and HYA_GA_ were higher (*p* < 0.05) than LP and HYA, respectively, as a consequence of GA encapsulation inside the bilayers of liposomes.

The particle size distribution (PSD) value can reflect the homogeneity of liposome and hyalurosome samples [24]. In our study, both kinds of vesicles showed a good PSD value, lower than 0.3, demonstrating that the extrusion could be a suitable process to obtain uniform samples [25].

Concerning the ζ potential, all the prepared formulations were characterized by negative ζ potential values, in agreement with other published works [26,27]. This result was owing to the presence of phosphatidylcholine, which is a zwitterionic molecule containing the phosphate and choline functional groups. At pH 7.4, phosphatidylcholine shows the prevalence of negatively charged phosphate groups imparting a negative value to the liposome surface [28]. On the other side, in the presence of sodium hyaluronate, an increase of negativity (*p* < 0.05) was observed owing to its anionic nature and the interaction between the polymer and the choline groups of phosphatidylcholine. This result was in agreement with other findings [29] and can represent a proof of the presence of sodium hyaluronate on the vesicle surface.

### 2.2. Vesicle Physical Stability

**Figure 1:**
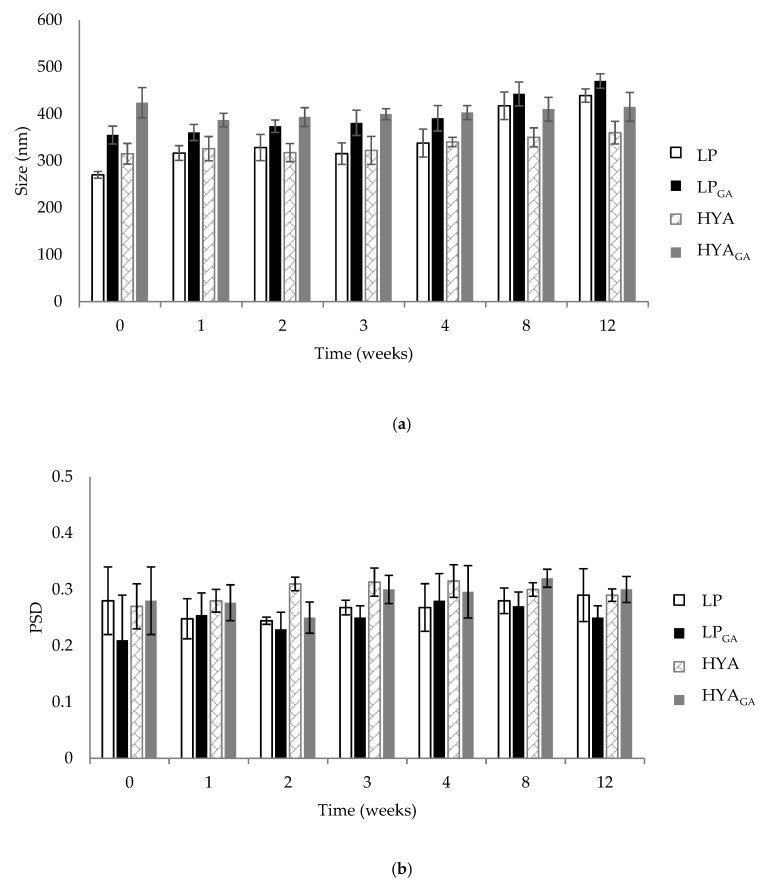
Variation of size (**a**) and particle size distribution (PSD) (**b**) of unloaded and loaded liposomes (LP) and hyalurosomes (HYA) during 12 weeks of storage at 4–8 °C (mean ± SD, *n* = 3). GA, glycyrrhetinic acid.

Physical stability of liposomes and hyalurosomes is an important factor to predict the quality of formulations [30]. To determine vesicle stability, changes of size and PSD were monitored over a period of storage at 4–8 °C for 12 weeks. The vesicle variation in terms of size and PSD is reported in Figure 1a,b, respectively. Liposomes maintained their size until 4 weeks; after this period, an increase in size was observed (*p* < 0.05), reaching, for LP and LP_GA_, a final diameter equal to 439 and 470 nm, respectively. No relevant changes with respect to size and PSD were detected for hyalurosomes, which showed a constant mean diameter and a homogeneous size distribution over the tested period. The higher stability of HYA and HYA_GA_ could be ascribed to the highly negative ζ potential that probably limited the phenomenon of aggregation and precipitation of the vesicles during the storage period. In fact, as reported in the literature, higher values of ζ potential favor higher electrostatic repulsion among the vesicles and, consequently, a greater stability [31]. Moreover, the highest stability of hyalurosomes was in agreement with other previous works, suggesting that the aggregation of vesicles containing sodium hyaluronate is limited by the immobilizing effect of the polymer [12,20].

### 2.3. Determination of Encapsulation Efficiency

Encapsulation efficiency (EE%) represents a critical parameter in the evaluation of the potentiality of a delivery system [14]. The EE% of GA was similar, with no statistical differences between LP_GA_ and HYA_GA_ (∼60%, Table 1), indicating the good ability of both vesicles to incorporate GA into the phospholipid bilayers. These EE% values were similar to those obtained in the work of Li and colleagues [14], in which conventional liposomes and elastic vesicles containing GA were prepared. The authors observed EE% values equal to 50.31± 4.21% and 73.12 ± 4.63% for conventional liposomes and elastic vesicles, respectively, and attributed the higher EE% of elastic vesicles to the presence of sodium deoxycholate that favors GA incorporation into the lipid bilayers. Regarding the presence of sodium hyaluronate, we did not observe any impact of the polymer on EE%. This finding was in agreement with results previously reported by Castangia et al. [12]. In this work, the authors prepared liposomes and hyalurosomes containing the liquorice extract, obtained by percolation in ethanol of *Glycyrrhiza glabra* L. roots, or the raw glycyrrhizin. The authors reported no significant differences in the EE% values of the liquorice extract and glycyrrhizin between liposomes and hyalurosomes.

### 2.4. In Vitro Release Studies from Liposomes and Hyalurosomes

**Figure 2:**
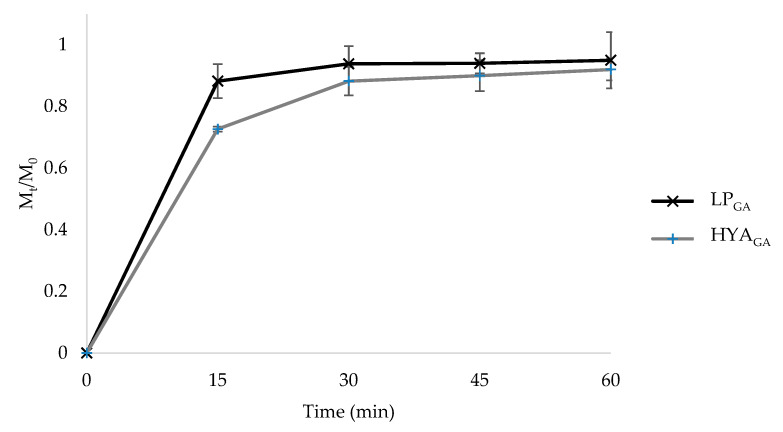
In vitro GA release in PBS/EtOH (7:3 *v/v*) from LP_GA_ and HYA_GA_ (mean ± SD, *n* = 3).

The effectiveness of a drug delivery system can be influenced by the release behavior of the active substance. Figure 2 shows the fractional amount of GA released from LP_GA_ and HYA_GA_ as a function of time. As can be seen from Figure 2, a fast release of GA was observed in PBS/EtOH (7:3 *v*/*v*), reaching 88% and 73% of the total GA amount after 15 min for LP_GA_ and HYA_GA_, respectively. At this time point, the difference observed in the value of GA released from LP_GA_ and HYA_GA_ (*p* < 0.05) was probably related to the presence of sodium hyaluronate on the vesicle surface, which influenced the GA partition between the vesicle bilayer and the release medium. However, after 30 min, the release was no longer considerably affected by the type of vesicles. Considering that the first hours are decisive for the wound healing process [32], the fast release of GA could be useful for reaching its maximum drug amount at the wound site immediately after vesicle application, thus being advantageous to accelerate the tissue repair healing.

### 2.5. Biocompatibility of LPGA and HYAGA

To assess the biocompatibility of GA-loaded vesicles, the viability of 3T3 fibroblasts was analyzed by MTT assay. It has been reported that GA can inhibit the proliferation of different cell lines [33]; therefore, it was investigated if 3T3 cell line viability was affected by GA itself, prior to testing LP_GA_ and HYA_GA_. Fibroblasts were treated with GA concentrations ranging from 3 to 20 μg/mL, corresponding to those contained in the vesicle suspensions tested for the MTT assay. The results reported in Table 2 show the absence of a toxic effect after treatment with GA, as viable population is superimposable to the control ones; cytotoxicity was rated based on cell viability relative to the control group, considering significant values below 95%.

**Figure 3:**
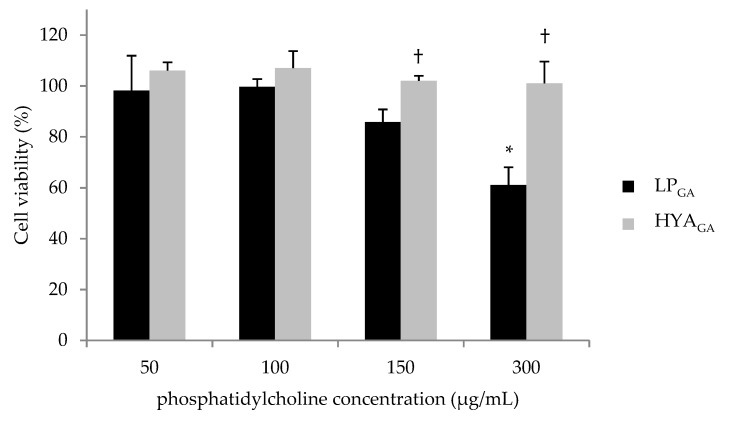
Viability of 3T3 cells after different dilutions of LP_GA_ and HYA_GA_ treatment for 24 h. Results are shown in mean percentage respect to the controls taken as 100% (mean ± SD, *n* = 3) Significance indicated by * *p* < 0.05 compared with the control and ^†^ *p* < 0.05 compared with LP_GA_.

Then, cells were treated with LP_GA_ or HYA_GA_ at different dilutions in order to obtain final concentrations of phosphatidylcholine and GA ranging from 50 to 300 μg/mL and 3 to 20 μg/mL, respectively (Figure 3). The obtained data revealed that both types of vesicles are highly biocompatible up to 150 μg/mL phosphatidylcholine concentration. Interestingly, HYA_GA_ did not exert any toxicity up to the maximum tested concentration of phosphatidylcholine (300 μg/mL). Moreover, HYA_GA_ showed a superior biocompatibility with respect to LP_GA_ when phosphatidylcholine concentrations were equal to 150 and 300 μg/mL (*p* < 0.05).

**Figure 4:**
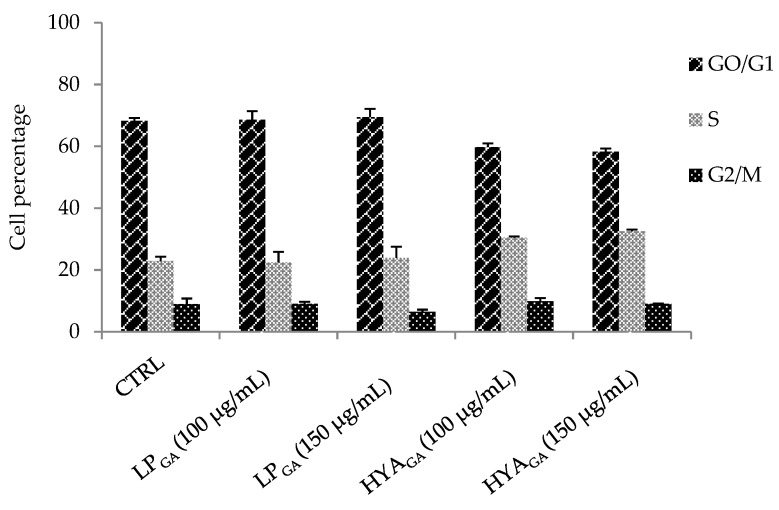
Cell cycle analysis of 3T3 cells treated with LP_GA_ or HYA_GA_ (100 or 150 µg/mL phosphatidylcholine concentration) for 24 h. The histograms report the percentage of cells in the different phases of the cell cycle, evaluated by flow cytometric assay.

To deeply investigate the activity of the vesicles under test on cell proliferation, cell cycle analysis was performed by means flow cytometry. The results showed the absence of significant alteration in cell cycle progression. Moreover, HYA_GA_ seem more promising again, as they are able to induce a moderate increase of cell percentage in S phase at both 100 and 150 μg/mL of phosphatidylcholine concentrations with respect to the controls; this could indicate a stimulating effect on fibroblast proliferation—useful and desirable in the contest of wound treatment (Figure 4). This result was in agreement with previous findings reporting the re-epithelising and tissue remodeling properties of sodium hyaluronate [12,20,34].

### 2.6. In Vitro Release Studies from the Final Dressings

The employment of dressings allowed to optimize the application of vesicle suspensions on skin wounds, to simplify self-medication, and consequently to increase patient compliance. Considering their ability to promote conformability to the wound area [35] and their physico-chemical properties, Spanish Broom, flax, and hemp dressings could be proposed as new biomaterials able to cover the wounds and to deliver at the wound site nanosystems carrying functional molecules such as GA.

Preliminary studies were conducted in order to evaluate the adsorption ability of the different dressings. For this purpose, Spanish Broom, flax, and hemp dressings (2 × 2 cm) were impregnated with different volumes of vesicle suspensions ranging from 0.15 to 1 mL. Impregnation of the dressings was considered completed after 15 min and the maximum volume efficiently adsorbed for all the dressings was found to be 0.5 mL. This behavior is in agreement with our previous work [6].

**Figure 5:**
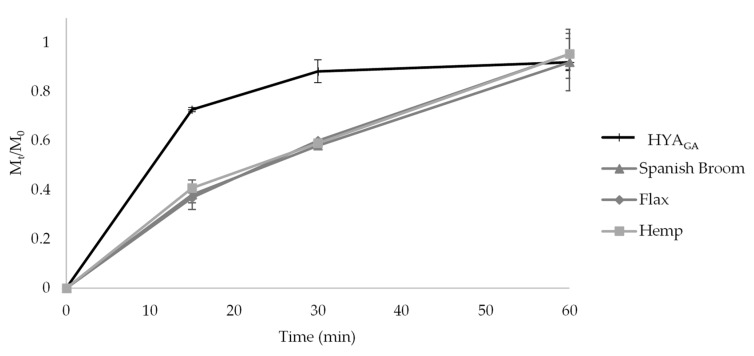
In vitro GA release in PBS:EtOH (7:3 *v/v*) from HYA_GA_ and from Spanish Broom, flax and hemp wound dressings loaded with HYA_GA_ (mean ± SD, *n* = 3).

Figure 5 shows the release profiles of GA obtained from HYA_GA_ and from the different dressings loaded with HYA_GA_ in PBS/EtOH (7:3, *v*/*v*). When HYA_GA_ were loaded into the dressings, a lower amount of GA was released in the first 30 min with respect to the free hyalurosome suspension HYA_GA_ (*p* < 0.05). This result could probably be related to the diffusion of GA in the dressing environment, in addition to GA partition between the vesicle bilayer and the release medium or the dressing. However, the complete release of GA was also obtained after 60 min for the final dressings. Similar release profiles were observed with dressings loaded with LP_GA_ (data not shown). These results indicated that, when vesicles were loaded into Spanish Broom, flax, and hemp dressings, the total GA amount loaded in the final formulations can be available at the wound site within 60 min from their application, thus favoring the wound healing process.

## 3. Materials and Methods

### 3.1. Materials

l-α-phosphatidylcholine from egg yolk, 18-β-glycyrrhetinic acid (GA), and all the solvents were purchased from Sigma-Aldrich (Milan, Italy). Sodium hyaluronate (molecular weight = 800–1200 kDa) was sourced from Farmalabor (Canosa di Puglia, Italy). Spanish Broom dressing was provided by Prof. Giuseppe Chidichimo, from University of Calabria (Arcavacata di Rende, CS, Italy). Flax and hemp dressings were obtained from Linificio e Canapificio S.r.l. (Villa D’Almè, Bergamo, Italy). All other chemicals were purchased from Carlo Erba (Milan, Italy). Mouse 3T3 fibroblast cells were from the American Type Culture Collection (ATCC; Rockville, MD, USA). All reagents for cell culture were obtained from Sigma-Aldrich (St. Louis, MO, USA), if not otherwise specified, and were ultrapure grade. Roswell Memorial Park Institute (RPMI-1640) medium, fetal bovine serum (FBS), and Dulbecco’s phosphate-buffered saline (DPBS) were purchased from Gibco-Life Technologies (Carlsbad, CA, USA). All plastic supports were purchased from Falcon, BectonDickinson (Franklin Lakes, NJ, USA). Phosphate buffer solution at pH 7.4 (PBS) was prepared with the following composition: 2.38 g/L Na_2_HPO_4_ × 12 H_2_O, 0.19 g/L KH_2_PO_4_, 8 g/L NaCl. For GA determination, a phosphate buffer with 9.15 g/L Na_2_HPO_4_ × 12 H_2_O, adjusted at pH 7.0 with H_3_PO_4_, was also prepared.

### 3.2. Liposome and Hyalurosome Preparation

Liposomes were prepared by the film rehydration method followed by extrusion, reported by Uchino et al. [36] with some modifications. Briefly, l-α-phosphatidylcholine (300 mg) was dissolved in a mixture of CHCl_3_-CH_3_OH (10 mL, 9:1 *v*/*v*) in a round-bottomed flask and, subsequently, the organic mixture was evaporated using a rotatory evaporator (Buchi Rotavapor R-200, Flawil, Switzerland) under reduced pressure (80 mbar) at 55 °C for 150 min. The dry lipid film was then hydrated with 40 mL of PBS for 1 h, obtaining a final phosphatidylcholine concentration equal to 7.5 mg/mL. The resulting suspension was extruded 10 times through a polycarbonate membrane with a pore size of 100 nm (LiposoFast manual syringe extruder, Avestin Europe GmbH, Mannheim, Germany) in order to reduce and homogenize vesicle size [25]. Hyalurosomes were obtained through rehydration of the lipid film with a sodium hyaluronate solution, prepared by dissolving the polymer in PBS (0.5 mg/mL) for 30 min under stirring at 200 rpm. For the preparation of the loaded vesicles, GA was solubilized in the organic phase at a concentration of 2 mg/mL. The different formulations were named on the basis of their composition as follows: LP and LP_GA_ for unloaded and loaded liposomes, respectively; HYA and HYA_GA_ for unloaded and loaded hyalurosomes, respectively.

### 3.3. Vesicle Characterization

Liposomes and hyalurosomes were characterized in terms of size, particle size distribution (PSD), and ζ potential. Size and PSD were measured by PCS (photon-correlation spectroscopy) using a Brookhaven 90-PLUS instrument (Brookhaven Instruments Corp., Holtsville, NY, USA) with He-Ne laser beam at a wavelength of 532 nm (scattering angle of 90°). Suspensions were diluted (1:500; *v*/*v*) in ultrapure water (18.2MΏ cm, MilliQ apparatus by Millipore, Milford, MA, USA). ζ potential measurements were carried out at 25 °C on a Malvern Zetasizer 3000 HS instrument (Malvern Panalytical Ltd., Malvern, UK), after the same dilution.

### 3.4. Vesicle Physical Stability

The physical stability of the prepared liposomes and hyalurosomes was assessed by monitoring the size and the PSD over 12 weeks of storage at 4–8 °C. For this study, aliquots of vesicle suspensions were diluted in ultrapure water (1:500; *v*/*v*) and the change of liposome and hyalurosome size and PSD index was measured using PCS.

### 3.5. Determination of Encapsulation Efficiency

To quantify the amount of GA not incorporated into the vesicles, a modification of the dialysis method previously described [37] was employed. Briefly, 3 mL of the liposome or hyalurosome suspensions were placed inside a Visking Tubo Dialysis membrane (Medicell International Ltd., London, UK) with a cut-off size of 14,000 Dalton. The tube was then immersed into 150 mL of PBS (external phase) and kept at 25 °C for 48 h under stirring at 100 rpm. The free GA, separated from liposomes or hyalurosomes and contained in the external phase, was detected via the HPLC method. The chromatographic system was composed of a Shimadzu (Milan, Italy) LC-10ATVP chromatographic pump and a Shimadzu SPD-10AVP UV–vis detector set at 250 nm. Separation was obtained on a Phenomenex (Torrance, CA, USA) Synergi Fusion-RP 80A (150 mm × 4.6 mm I.D., 5 µm) coupled to a Phenomenex (Torrance, CA, USA) SecurityGuard C18 guard cartridge (4 mm × 3.0 mm I.D., 5 µm). The mobile phase was a mixture of an aqueous phosphate buffer at pH 7.0 and acetonitrile (40:60, *v/v*). The flow rate was 0.4 mL/min and manual injections were made using a Rheodyne 7125 injector with a 20 µL sample loop. Data processing was handled by means of a CromatoPlus computerized integration system (Shimadzu Italia, Milan, Italy). The calibration curve of concentration versus peak area ratio was plotted at a concentration range of 0.1–5 µg/mL and a good linearity was found (*R*^2^ = 0.998).

The encapsulation efficiency (EE) was calculated using the following equation:

EE% = (Total amount of GA−Amount in the external phase) × 100/Total amount of GA(1)

### 3.6. In Vitro Release Studies from Liposomes and Hyalurosomes

For this study, vesicle suspensions (0.5 mL) were placed in a beaker containing 10 mL of PBS and ethanol (7:3 *v*/*v*) mixture, and then the system was put in a water bath at 32 °C with an agitation speed of 100 rpm. At predetermined time intervals, aliquots of the sample were taken from the medium, centrifuged at 14,500 rpm for 15 min, and the supernatant was analyzed through HPLC [25]. The release of GA over the time was determined as M_t_/M_0_ (fractional amount), where M_t_ represents the cumulative amount of GA released at each time and M_0_ the total GA mass into the vesicle suspensions.

### 3.7. Cell Culture and Treatment

3T3 fibroblasts were maintained in RPMI 1640 medium, supplemented with 10% FBS, 2 mM l-glutamine, 100 U/mL penicillin, and 100 μg/mL streptomycin, at 37 °C and 5% CO_2_. Cells were seeded at 1 × 10^4^ cells/cm^2^ in plastic wells and allowed to grow for one day before treatment. Specifically, cells were treated without (control) or with GA (dissolved in a mixture of PBS/EtOH 97:3 *v*/*v*) at different concentrations ranging from 3 to 20 μg/mL. Loaded vesicles were tested at different dilutions corresponding to 50–300 μg/mL and 3–20 μg/mL concentration range of phosphatidylcholine and GA, respectively.

#### 3.7.1. Cell Viability Assay (MTT)

To assess the in vitro toxicity of GA and of loaded vesicles, 3T3 cells seeded in 24-well culture plates were treated for 24 h. MTT (3-(4,5-dimethylthiazol-2-yl)-2, 5-diphenyl tetrazolium bromide) stock solution was prepared as 2.5 mg/mL in PBS. Then, 0.02 mL of this solution was added to each well, and cells were incubated for four hours. Next, the supernatant was removed and the dark blue formazan crystals were dissolved in 1 mL of isopropanol. The absorbance at 570 nm (which reflects the relative viable cell number) was determined by UV–vis spectrophotometer (Kontron Uvikon 860, Augsburg, Germany).

#### 3.7.2. Cell Cycle Analysis

DNA profiles of 3T3 cells were obtained by flow cytometry according to Erba et al. [38]. Cells treated with LP_GA_ and HYA_GA_ at the indicated dilutions for 24 h, were detached with 0.25% trypsin–0.02% EDTA solution and counted. Then, 1 × 10^6^ cells were washed from growth medium and centrifuged at 240 × g for ten minutes. The pellet was suspended in 1 mL of a solution containing 0.1% trisodium citrate, 0.01% Igepal, 10 μg/mL of RNAse, and 50 μg/mL of propidium iodide (PI). After 30 min at 37 °C in the dark, the isolated nuclei were analyzed by using a Bryte HS flow cytometer (Bio-Rad, Hercules CA, USA) equipped with a Xe/Hg lamp and a filter set to obtain an excitation at 488 nm. PI fluorescence was collected on a linear scale at 600 nm, and the DNA distribution was analyzed by the ModFit 5.0 software (Verity, Topsham, ME, USA).

### 3.8. Preparation of Wound Dressings

#### 3.8.1. Impregnation of Spanish Broom, Flax, and Hemp Dressings with Vesicle Suspensions

The final wound dressings were prepared using three different supporting materials, Spanish Broom, flax, and hemp. Spanish Broom fibers were extracted by patented DiCoDe (digestion–compression–decompression) process [39], which provided fibers with a high cellulose content (91.7 ± 0.1%), excellent mechanical properties (tenacity 35.9 ± 1.6 cN/tex, strain at break 5.8 ± 1.7%), and good cyto-compatibility [7]. Flax and hemp cellulose content was 75.3± 0.3% and 70%, respectively [6,40].

In order to obtain the final wound dressings, vesicles prepared, as reported in Section 3.2 were absorbed onto Spanish Broom, flax, and hemp dressings by impregnation [7], the most commonly used and convenient process for depositing nanosystems on fibers [41]. Preliminary studies were conducted in order to investigate dressing ability to adsorb vesicle suspensions. Specifically, Spanish Broom, flax, and hemp dressings were cut into 2 × 2 cm pieces and different volumes of vesicle suspensions, ranging from 0.15 to 1 mL, were deposited onto these samples. The ability of dressings to adsorb the vesicle suspension was evaluated for 1 h. The developed dressings were then sealed in aluminum foil bags and stored at 4–8 °C until use.

#### 3.8.2. In Vitro Release Studies from Wound Dressings

The different dressings, obtained as reported in Section 3.8.1, were placed in 10 mL of a mixture of PBS and ethanol (7:3 *v*/*v*) and maintained at 32 °C under agitation (100 rpm). At predetermined time intervals, aliquots of the sample were taken from the medium and analyzed through HPLC after centrifugation, as reported in Section 3.6.

### 3.9. Statistical Analysis

All the experiments were performed in triplicate. The results were expressed as mean ± standard deviation (S.D.). For all the performed studies, Student’s *t*-test was used to determine statistical significance. Differences were deemed significant for *p* < 0.05.

## 4. Conclusions

In this study, Spanish Broom, flax, or hemp dressings for skin wound treatment were prepared by impregnation with liposomes or hyalurosomes loaded with GA, previously prepared through the film hydration method followed by extrusion. Liposomes and hyalurosomes differed for the presence of sodium hyaluronate, selected on the basis of its well-known role in skin wound healing. Liposomes and hyalurosomes of nanometric size and homogeneously dispersed were successfully prepared and efficiently loaded with GA. Hyalurosomes showed appropriate stability during the storage period, which can be correlated to the negative ζ potential, and allowed a fast and complete release of GA. Moreover, hyalurosomes were not cytotoxic on fibroblast up to higher concentration with respect to the liposomes. Both types of vesicles did not significantly impair cell cycle progression, but a moderate increase of S phase cell percentage was observed after treatment with hyalurosomes, suggesting a stimulating effect on fibroblast proliferation—useful and desirable in the contest of wound treatment. Finally, GA was completely released within 60 min from the different dressings impregnated with hyalurosomes. Considering that the first hours are decisive for the wound healing process, this result could be promising to accelerate the skin repair. In conclusion, this study suggests that Spanish Broom, flax, or hemp materials impregnated with GA-loaded hyalurosomes could be considered new functional dressings for the treatment of skin wounds, even if only future in vivo studies will be able to definitely confirm this hypothesis.

## Figures and Tables

**Table 1:** Size (nm), particle size distribution (PSD), ζ potential (mV), and encapsulation efficiency (EE %) of the different vesicles. HYA, hyalurosome; LP, liposome; GA, glycyrrhetinic acid.

	Size (nm)	PSD	ζ Potential (mV)	EE%
LP	270 ± 7	0.28 ± 0.06	−47.96 ± 1.45	/
LP_GA_	355 ± 19	0.21 ± 0.08	−46.72 ± 1.91	58.62 ± 3.25
HYA	315 ± 22	0.27 ± 0.04	−55.30 ± 1.43	/
HYA_GA_	424 ± 32	0.28 ± 0.06	−56.90 ± 0.48	59.22 ± 8.18

**Table 2:** Cell viability of 3T3 cells after culture with different concentrations of GA for 24 h. Results are shown in mean percentage respect to the controls taken as 100% (mean ± SD, n = 3).

	GA Concentration (μg/mL)			
	3.4	6.7	10	20
Viable cells (%)	91.9 ± 7.6	88.0 ± 8.5	88.6 ± 6.7	95.4 ± 6.6
